# Motor activation in literal and non-literal sentences: does time matter?

**DOI:** 10.3389/fnhum.2013.00202

**Published:** 2013-05-17

**Authors:** Cristina Cacciari, Francesca Pesciarelli

**Affiliations:** Department of Biomedical, Metabolic and Neurological Sciences, University of Modena and Reggio-EmiliaModena, Italy

**Keywords:** motion verbs, non-literal language, abstract meaning, motor activation

## Abstract

Despite the impressive amount of evidence showing involvement of the sensorimotor systems in language processing, important questions remain unsolved among which the relationship between non-literal uses of language and sensorimotor activation. The literature did not yet provide a univocal answer on whether the comprehension of non-literal, abstract motion sentences engages the same neural networks recruited for literal sentences. A previous TMS study using the same experimental materials of the present study showed activation for literal, fictive and metaphoric motion sentences but not for idiomatic ones. To evaluate whether this may depend on insufficient time for elaborating the idiomatic meaning, we conducted a behavioral experiment that used a sensibility judgment task performed by pressing a button either with a hand finger or with a foot. Motor activation is known to be sensitive to the action-congruency of the effector used for responding. Therefore, all other things being equal, significant differences between response emitted with an action-congruent or incongruent effector (foot vs. hand) may be attributed to motor activation. Foot-related action verbs were embedded in sentences conveying literal motion, fictive motion, metaphoric motion or idiomatic motion. Mental sentences were employed as a control condition. foot responses were significantly faster than finger responses but only in literal motion sentences. We hypothesize that motor activation may arise in early phases of comprehension processes (i.e., upon reading the verb) for then decaying as a function of the strength of the semantic motion component of the verb.

## Introduction

A consistent bulk of evidence showed that the motor schemata associated with action words are embedded in the corresponding cortical representations (for overviews, see Mahon and Caramazza, 2005, 2008; Pulvermüller, 2005; Willems and Hagoort, 2007; Kemmerer and Gonzalez-Castillo, 2010). The neural architecture of language-induced *motor resonance* would therefore comprise regions encoding information that reflects the sensory-motor properties associated with the underlying concept. Motor and premotor sites engaged in the production of actions would also be involved in the comprehension of action-related words and sentences in somatotopically consistent ways (Hauk and Pulvermüller, 2004; Pulvermüller et al., 2005, 2009; Tettamanti et al., 2005; but see Fernandino and Iacoboni, 2010; Boulenger et al., 2012). In sum, word and sentence processing would be grounded in the brain systems that underlie action and perception (Barsalou, 1999, 2008; Pulvermüller, 2005).

However, despite the impressive, and hard to summarize, amount of studies that favors the composite *Embodied and Grounded Cognition approach* (for overviews, see Mahon and Caramazza, 2005, 2008; Borghi and Cimatti, 2010; Pulvermüller and Fadiga, 2010; Weiskopf, 2010; Dove, 2011; Willems and Casasanto, 2011) important questions remain unsolved. For instance, it is still disputed whether motor activation arises in early phases of language comprehension (Pulvermüller, 2005; Zwaan and Taylor, 2006; Kaschak and Borreggine, 2008; Boulenger et al., 2012), due to automatic activation of the same neural circuitry for action and language-mediated action simulation, or later on (Boulenger et al., 2009; Papeo et al., 2009) reflecting late merging of information pertaining to the semantic and action systems (Mahon and Caramazza, 2008). Then, although many studies showed that motor systems become active when action-related words are comprehended, it still remains unclear whether motor systems activation is necessary for understanding those words when presented in isolation or in linguistic contexts (for a discussion, see Mahon and Caramazza, 2008; Shebani and Pulvermüller, 2013). As Willems and Casasanto (2011) recently put it, *the available evidence weights against the view that merely perceiving a perception or action word necessarily activates perceptuo-motor areas* (Pulvermüller, 2005) *while showing that these areas can be activated* (p. 7). Turning now to the problem at issue in the present study, it is still debated the extent to which the comprehension of non-literal motion sentences engages the same neural networks recruited when motion is conveyed by literal language (e.g., Aziz-Zadeh et al., 2006; Chen et al., 2008; Boulenger et al., 2009, 2012; Cacciari et al., 2011; Desai et al., 2011). Typically, non-literal sentences containing action-related verbs convey abstract meanings. For instance, when someone says *The employee runs the risk of being fired*, or *The rumor flew across town*, it is evident that she did not refer to concrete actions. Explaining abstract meanings in terms of embodied/grounded cognition has become particularly challenging: *Abstract concepts pose a classic challenge for grounded cognition. How can theories that focus on modal simulation explain concepts that do not appear modal?* (Barsalou, 2008, 634). One possibility is to assume, as Barsalou (2008) recently suggested that linguistic information may be more relevant for abstract than for concrete concepts. This would lead to a dual system (embodied for concrete meanings and disembodied for abstract domains), a claim that recently has been extensively discussed also because of its resemblance with the Dual Code theory proposed decades ago by Paivio (1986) (for a discussion, see Kousta et al., 2010; Borghi et al., 2011; Dove, 2011; Willems and Casasanto, 2011). One way to reconcile the embodied and disembodied views on linguistic meanings is to assume the existence of multiple representations associated to words originating from perception/action, social and linguistic domains (Borghi and Cimatti, 2010, p. 2): *similarly to real tools, words can be considered as instruments to act in social words, thus as social words. (…) due to a different acquisition process, the role played by actions performed through words—by linguistic information—is more relevant for abstract than for concrete words*. Along similar lines, Kousta et al. (2010) proposed that concrete and abstract concepts may bind different types of information: experiential information (sensory, motor, and affective) and also linguistic information. While sensory-motor information would be more preponderant for concrete concepts, affective information would play a greater role for abstract concepts. In sum, claiming that abstract words may be predominantly processed in the language system and concrete words in sensory-motor systems to a larger extent (e.g., Kousta et al., 2010; Borghi et al., 2011; Scorolli et al., 2011) would confirm that *our concepts are not merely couched in sensorimotor representations but also in linguistic representations (words, phrases, sentences)*. (Dove, 2011, 7). The idea that perception-action, linguistic and social information are more relevant for abstract than for concrete words mitigates, if not disconfirms, one the tenets of the Embodied view that all cognition is grounded in bodily states, modal simulations and situated actions (for a discussion, see Borghi and Cimatti, 2010; Kousta et al., 2010; Dove, 2011; Willems and Casasanto, 2011).

Motion verbs can be used in different ways that depend on the linguistic information surrounding the action verb. For instance, in *The man runs in the beautiful country* the motion verb conveys an actual change of location of an animate subject. In contrast, in *The road runs along the impetuous river* there is no reference to a physical entity moving: this sentence in fact conveys a *fictive motion* (Talmy, 2000). Typically, fictive motion sentences express a spatial relation between a path (or linear event) and a landmark (Talmy, 2000; Matlock, 2004; Wallentin et al., 2005; Richardson and Matlock, 2007). An inanimate subject (e.g., *road*, *railway*) is coupled with a motion verb to convey a static meaning. Are fictive sentences literal or figurative statements? As Jackendoff and Aaron (1991) claimed, fictive motion sentences are one way to ordinarily refer to space or locations: *there is no way to express spatial extent other than by using such expressions. … virtually all the extent verbs of English can also be used as motion verbs* (p. 329). A simple test may further clarify the issue: while it would be odd to say *Metaphorically speaking, the road goes from Los Angeles to New Mexico*, it makes perfect sense to say *Metaphorically speaking, the woman runs with her fantasy often*. Hence, following Jackendoff and Aaron, we propose to consider fictive sentences as literal rather than figurative statements. Motion verbs can be used in two further ways: they can be inserted in metaphorical statements as, for instance, in *The rumor flew across town*, or *The woman runs with her fantasy often*. In these cases motion verbs do not take their default argument in the subject or object position. In the metaphorical sense, motion verbs are used at a higher level of abstraction to refer to any instance of goal-driven conjoint motion. In this view, the metaphorical use of a motion verb preserves the semantic component of motion (Torreano et al., 2005; Cacciari et al., 2010, 2011). Lastly, a motion verb can be part of an idiom string as, for instance, in *The new employee walks the chalk line*, or *Between the neighbors runs bad blood*. While literal motion sentences convey an actual movement and metaphorical sentences an abstract motion, in idiom strings the semantic motion component of the verb typically vanishes because of the conventionality, arbitrariness of the relationship between the idiom constituent words and the global figurative meaning.

The picture on the involvement of motor regions in the comprehension of action verbs that convey actual or abstract actions is rather complex. In what follows, we briefly examine the studies that shed more light on this issue. In the study that led Glenberg and colleagues to propose the *Action Compatibility Effect* (ACE, Glenberg and Kaschak, 2002; Glenberg et al., 2008), participants judged whether a sentence was or not meaningful (sentence sensibility task) when the meaning conveyed the transfer of a concrete object or abstract information. Reaction times were faster when the action conveyed by the sentence matched the action required to respond in both concrete and abstract sentences. Turning to TMS studies, Oliveri et al. (2004) showed that action-related verbs and nouns elicited greater activation in the primary motor cortex than non-actions stimuli. Differently, Buccino et al. (2005) and Glenberg et al. (2008) observed motor excitability without any difference between abstract and action-related sentences. Other studies obtained different if not opposite findings: Papeo et al.'s study (2009) showed no specific involvement of the left primary motor cortex in early and mid time windows (i.e., 170 and 350 ms after stimulus presentations) but only later on, namely 500 ms after presentation of hand-action verbs. The literature highlighted the presence of further constraints on motor excitability. For instance, in Papeo et al. (2011) motor cortex was found active when hand-related action verbs were expressed in first person but less so, or not at all, with a third person form. Tomasino et al. (2007) observed activation of M1 only when participants were explicitly asked to perform an explicit mental simulation of the verb content. In Cacciari et al. (2011) the literal or non-literal context in which motion verbs occurred modulated motor excitability: in fact the MEPs response was largest with literal sentences, followed by fictive sentences and metaphorical motion sentences. No motor excitability occurred in idiomatic sentences disconfirming Boulenger et al.'s (2009) claim of activation of motor cortices for idiomatic sentences. However, in Boulenger et al.'s fMRI study motor activation occurred at a time window later than that of the TMS stimulation in Cacciari et al. (2011; see also Papeo et al., 2009). Finally, in Cacciari et al. (2010) motor sentence fragments (formed by a NP followed by a motion verbs) elicited a significant change in the MEPs amplitude but only when the sentential subject was animate (i.e., in *The lady runs* but not in *The highway runs*).

Several fMRI studies were conducted as well to elucidate the neural links between language and action systems. But again, the resulting picture is far from homogenous (e.g., Aziz-Zadeh et al., 2006; Tomasino et al., 2007; Bedny et al., 2008; Chen et al., 2008; Boulenger et al., 2009; Raposo et al., 2009; Fernandino and Iacoboni, 2010; Bedny and Caramazza, 2011). A recent MEG study of Boulenger et al. (2012) seems to provide evidence of an early automatic activation of motor areas for idiomatic as well as literal sentences. Very early on (i.e., 150–250 ms after the final literal/idiomatic disambiguating word) brain regions as the temporal pole, dorsolateral prefrontal cortex and Broca's region were found to be differentially activated by literal and idiomatic sentences. Early activation in the motor system at the same early latencies (150–250 ms onward) suggested that motor schemata were activated regardless of the idiomatic or literal nature of the sentence. However, many of the idiom strings also had a plain literal meaning, therefore one has to assume that meaning dominance led participants to interpret ambiguous idiom strings as idiomatic rather than literal, which cannot be taken for granted. Then, the extremely scarce presence of non-action sentences, together with a 50% of idiomatic sentences, may have led participants to develop specific processing strategies.

In the present study we further explored the presence of motor activation in the comprehension of literal and non-literal sentences containing motion verbs. We used a behavioral task (sensibility judgment) used in many previous studies and the same set of controlled literal, metaphorical, idiomatic, fictive motion sentences and mental sentences of the TMS study above mentioned (Cacciari et al., 2011). In contrast with recent evidence (Boulenger et al., 2009, 2012) but consistently with, for instance, Raposo et al. (2009) and Aziz-Zadeh et al. (2006), in Cacciari et al. (2011) we did not observe motor activation for idiomatic sentences. This lack of motor activation in idiomatic motion sentences was attributed to the fact that when the motion verb is embedded in an idiom string, it loses any perceivable semantic trace of action because of the arbitrary relationships between literal and idiomatic meaning. Differently from idioms, metaphors maintain the original meaning of the constituent words and, more importantly motion, they preserve the motion component of the verb as literal sentences: in both cases a motion is implied, but in the metaphorical sense the motion verb is used at a higher level of abstraction to refer to any instance of goal-driven conjoint motion. Despite the fact that many idioms originate from metaphors, this origin is often lost and unperceived by readers. As Aziz-Zadeh et al. (2006) noted, *it is possible that once a metaphor is learned, it no longer activates the same network that it may have initially. That is, although a metaphor like “grasping the situation” when first encountered may have utilized motor representations for its understanding, once it is overlearned it no longer relies on those representations*.

However, there may be alternative ways for explaining the lack of motor activation in idiomatic sentences. To begin with, in our TMS study the sentences were presented in three separate segments: first the noun phrase, then the verb, and finally the sentence completion that clarified the literal vs. figurative nature of the sentence (e.g., *Diego/cammina/sul filo del rasoio spesso*/, *Diego/walks/on the edge of the razor often/*). This raises the possibility that participants may not have had time enough to revise the literal interpretation assigned to the first two parts of the sentence and to process the idiomatic meaning of the sentence prior to the TMS stimulation (occurring just at the end of the sentence). As Boulenger et al. (2012) noted, while the semantic space explored while comprehending literal sentences is narrower, it can be more demanding for idiomatic sentences as a wider semantic space has to be searched. Moreover, idiom comprehension requires at the same time compositional and non-compositional processing: in fact idioms are understood by composing the ordinary meanings of the words until the idiomatic nature of the string is recognized, then the corresponding idiom configuration is retrieved from semantic memory and its meaning integrated in the sentential meaning (Cacciari and Tabossi, 1988). Hence processing idioms may be more resource and time consuming than corresponding literal sentences.

To explore the potential effects of these factors, we designed the present study in which participants judged the sensibility (i.e., meaningfulness) of the same sentences used in the TMS study but presented in their full form and without a time limit. Participants judged sentence sensibility pressing a button with a hand finger or with a foot (action-congruent vs. incongruent effector). Motor activation is known to be sensitive to the action-congruency of the effector used for responding (Glenberg and Kaschak, 2002). As de Lafuente and Romo (2004) put it, reading words conveying foot-based motion may *make the motor homunculus move its feet*. All other things being equal, any significant difference between the responses emitted with an action-congruent vs. action-incongruent effector (in our case, foot vs. hand) may be interpreted as implying motor activation. We used leg-related motor verbs. It would have been interesting to also use hand-related verbs in order to have the ideal symmetric case. However, this was impossible for fictive motion sentences since by definition (Talmy, 2000) this type of sentence uses motion verbs conveying a change of space along a path or a change of location. Previous studies (Glenberg and Kaschak, 2002; Glenberg et al., 2008) found that effector congruency produced facilitation in response times. Since in this study we used leg-related action verbs, foot responses should be faster than hand responses. However, Boulenger et al. (2006; see also Buccino et al., 2005) recently reported that language appears to interfere with the motor system. Interference would occur particularly when sensorimotor and linguistic information are difficult to integrate and/or are temporally overlapping. So the exact direction of the effector congruency effect (facilitation vs. interference) is still under scrutiny.

The task of judging whether a sentence meaning is sensible or not responding with action-congruent vs. incongruent effectors is widely used in the Embodied cognition literature (for a review, see Fischer and Zwaan, 2008) since this task is considered as particularly apt to detect motor system activation (Fischer and Zwaan, 2008). This task has the advantage that it leaves full time to participants for processing the sentential meaning as compared to our 2011 TMS study where brain stimulation occurred just at the end of the sentence. Comprehension unfolds in time, hence dividing the sentence into three fragments (NP, verb, sentence completion), presented one at a time for a given lag, as in our TMS study, may have required subjects to recompute the sentence meaning assigned after the second fragment when the arrival of the final segment made clear that the sentence was non-literal. It is well-known that recomputing a sentential meaning requires time and resources. Hence presenting the entire sentence has the advantage to eliminate the need of recomputing the non-literal meaning at the end of the sentence. Then, if motor activation requires more time to emerge in idiomatic motion sentences, due to meaning reinterpretation processes and to the more demanding nature of idiom understanding (Boulenger et al., 2012), leaving more time to participants, as it is the case with the sentence sensibility task, may led to motor activation not only in literal, fictive and metaphorical motion sentences, as in our TMS study, but also in idiomatic sentences.

## Experiment

### Materials and methods

We used the same controlled experimental materials of Cacciari et al. (2011) adding a motor and a mental verb to the list to have an equal number of stimuli per condition. This led to twenty-eight familiar Italian verbs expressing a leg-related movement (e.g., *run*, *walk*, *escape*, *cross*, *go*). The effector congruency of the motion verbs was tested in the norming phase of Cacciari et al. (2010) by asking five subjects to determine the effector mainly used to perform the action conveyed by each verb. There were four types of sentence for each of the 28 motion verb: (1) Literal motion sentences (e.g., *The man runs in the beautiful country*); (2) Metaphorical motion sentences (e.g., *The woman runs with her fantasy often*); (3) Idiomatic motion sentences (e.g., *Between the neighbors runs bad blood*); (4) Fictive motion sentences (e.g., *The road runs along the impetuous river*). Twenty-eight sentences of similar length and syntactic structure containing a mental verb acted as control sentences (e.g., *Cristina considers the idea very interesting*). This led to 140 experimental sentences (see Appendix for examples). The five types of sentence had the same verbal tense, they were all in a third-person form and had animate sentential subjects (with the exception of fictive sentences and three metaphorical sentences). One hundred and forty non-sensible sentences of similar length and structure were also created (e.g., *The fisherman shouts in a traffic light*; *He receives candles for a vegetable soup*). The lack of a semantically well-formed meaning was assessed asking 10 participants to judge whether the sentence had or not a sensible meaning on a 7-point scale (ranging from 1: The sentence is meaningless to 7: The sentence has a clear meaning) (*M* = 1.33, *SD* = 0.89).

The psycholinguistic characteristics that are known to affect comprehension latencies were controlled as well (see Table 1). The Age of Acquisition and the written frequency (COLFIS; Bertinetto et al., 2005) of each mental verb were matched to those of the paired motion verb. A written booklet containing literal, metaphorical, fictive, and idiomatic motion sentences was presented to 20 participants (different from those involved in the experiment) who were asked to assign a concreteness rating to the sentential meaning (from 0%: no concrete action at all, to 100%: totally concrete action). Basically, literal sentences were judged as conveying a concrete action (mean = 96.7%, *SD* = 4.0%) and much less so (or barely so) the other types of sentence. An additional group of 20 subjects was asked to determine the extent to which each sentence conveyed a literal or non-literal meaning using a 7-point scale (from 1: Literal meaning, to 7: Non-literal meaning). While the literalness of literal and mental sentences did not differ, metaphorical, fictive, and idiomatic motion sentences were judged as more figurative than mental sentences. Metaphorical motion sentences were judged as more figurative than fictive sentences but as figurative as idiomatic ones. In turn, idiomatic motion sentences were considered more figurative than fictive ones. A different group of 20 participants was asked to rate the comprehensibility of the sentences on a 7-point scale (from 1: Not at all comprehensible, to 7: Fully comprehensible). All sentences were highly comprehensible (mean = 6.1, *SD* = 0.5, range = 5.7–6.7) with literal motion sentences slightly but significantly more comprehensible than metaphorical, fictive and idiomatic ones but as comprehensible as mental sentences. The mean comprehensibility of metaphorical, fictive, idiomatic and mental sentences did not differ. The mean number of words in the five sentence types was balanced (mean = 7.5, *SD* = 0.1, range = 7.4–7.6).

**Table 1:** **Mean concreteness, written frequency, comprehensibility of the sentences, familiarity and semantic transparency of the idioms**.

**Type of subject**	**Literal**		**Metaphorical**		**Fictive**		**Idiomatic**		**Mental**	
	**Proper name**	**NP**	**Proper name**	**NP**	**Proper name**	**NP**	**Proper name**	**NP**	**Proper name**	**NP**
	**15**	**13**	**12**	**16**	**0**	**28**	**7**	**21**	**21**	**7**
**TYPE OF SENTENCE**										
Written frequency of the verb	236.7 (389)						211.7 (354)	
Figurativeness	2.0 (0.4)		5.6 (0.3)		3.7 (0.4)		5.2 (0.3)		2.1 (0.5)	
Number of words	7.4 (0.5)		7.5 (0.6)		7.5 (0.8)		7.6 (0.8)		7.4 (0.5)	
Sentence concreteness	96.7% (4.0)		3.1% (5.8)		25.4% (17.2)		6.4% (9.9)		–	
Sentence comprehensibility	6.5 (0.6)		5.7 (0.8)		5.9 (0.6)		5.7 (0.7)		6.7 (0.4)	
Semantic transparency	–						4.4 (1.2)		–	
Idioms familiarity	–						4.9 (0.3)		–	

*Standard deviations are reported in parentheses*.

In sum, the sentences were balanced for length and constituent words frequency and had high comprehensibility scores. We also controlled how much the idiom meaning was known (idiom familiarity), and how much the meaning of the idiom constituent words contributed to the figurative meaning (semantic transparency) (see Table 1). We asked 21 additional participants to rate each idiom on two separate rating scales (from 1: Unfamiliar idiom/Individual words do not contribute at all, to 7: Totally familiar idiom/Individual words contribute very much). The idioms were all familiar (mean = 4.9, *SD* = 0.34) and moderately transparent (mean = 4.4, *SD* = 1.2) with a between-idiom variability (range = 2.03–6.85) typical of this metalinguistic judgment.

### Participants

Forty eight students of the University of Modena-Reggio Emilia (33 female; mean age = 25.1 years, *SD* = 4.2) volunteered to participate. All were native speakers of Italian, had normal or corrected-to-normal vision and came from the same geographical area. None of the participants reported a history of prior neurological disorder. All participants were informed of their rights and gave written informed consent for participation in the study. The research was carried out fulfilling ethical requirements in accordance with standard procedures at the University of Modena-Reggio Emilia.

### Procedure

Participants were tested individually in a sound-attenuated room and sat at a distance of approximately 65 cm from the computer screen. The experimental instructions were presented on the screen and then repeated by the experimenter after the training session. Each trial began with a fixation cross (+) in the center of a computer screen. A spacebar press initiated the presentation of the sentence that was written in GENEVA BOLD 14 and appeared in the center of the screen. The sentences were divided into four lists, each list contained seven sentences per condition (literal, metaphorical, idiomatic, fictive motion, mental sentences) using a different verb so that participants were presented with each motion verb only in one experimental condition. As commonly done in the figurative language processing literature, but unfortunately often not in the Embodied language literature, figurative motion sentences (i.e., idiomatic and metaphorical) represented only 27% of sensible sentences to prevent participants from developing specific processing strategies. Fifty two meaningless sentences and 17 filler sentences with a well-formed literal meaning (without any motion verb) were added to the 35 experimental sentences forming each list so that each participant was presented with an equal number of sensible and non-sensible sentences.

Participants were randomly assigned to one of the four lists. The sentences were presented in four different blocks that differed as to the effector (hand finger vs. foot) with which participants were instructed to respond. The order of the blocks (e.g., Block 1: Hand response; Block 2: Foot response; Block 3; Hand response; Block 4: Foot response) was changed every four participants. In the Hand blocks, participants were instructed to press a *YES* button with their dominant finger as quickly and accurately as possible when the sentence was sensible and a *NO* button when the sentence was non-sensible. In the Foot blocks, participants were instructed to press a *YES* button pedal with their dominant foot as quickly and accurately as possible when the sentence was sensible and a *NO* button pedal when the sentence was non-sensible. The positions of the response buttons were counterbalanced across participants. Participants judged the sentence sensibility responding with the hand finger for half of the sentences and with the foot for the remaining. Hand and foot dominance were controlled using the *Lateral Preference Inventory* (Coren, 1993). The left hand was dominant in three participants and the left foot in three participants. A response deadline of 3000 ms was employed. Before the experiment, each participant performed 12 practice trials formed by sentences without any motion verb, half with sensible and half with non-sensible meanings. To be sure that participants knew the meaning of idiomatic sentences, at the end of the experiment they were presented with the list of idiomatic motion sentences and were asked to write down the sentence meaning. A rating of 0 was assigned to the answer *I do not know* or to a wrong meaning, 1 to a partially correct meaning and 2 to the correct meaning. The results (mean = 1.7, *SD* = 0.3, range = 1.3–2) suggest that participants indeed knew the idiom meanings.

Stimulus presentation and response collection were performed using a purpose-written E-Prime script (Psychology Software Tools).

### Results

**Figure 1:**
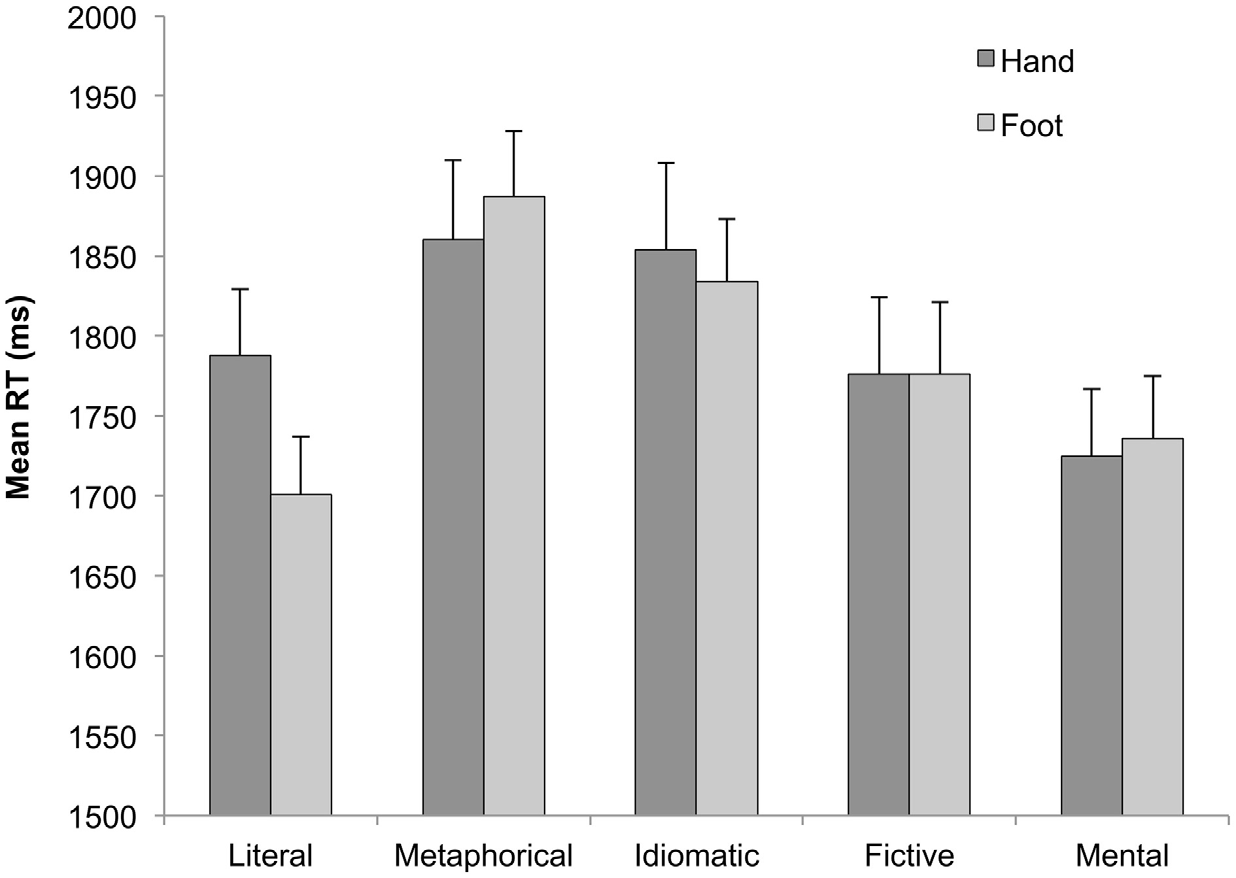
**Mean reaction times for responses emitted with hand (dark gray bar) and foot (bright gray bar) effectors in literal, metaphorical, idiomatic, fictive motion, and mental sentences**.

**Figure 2:**
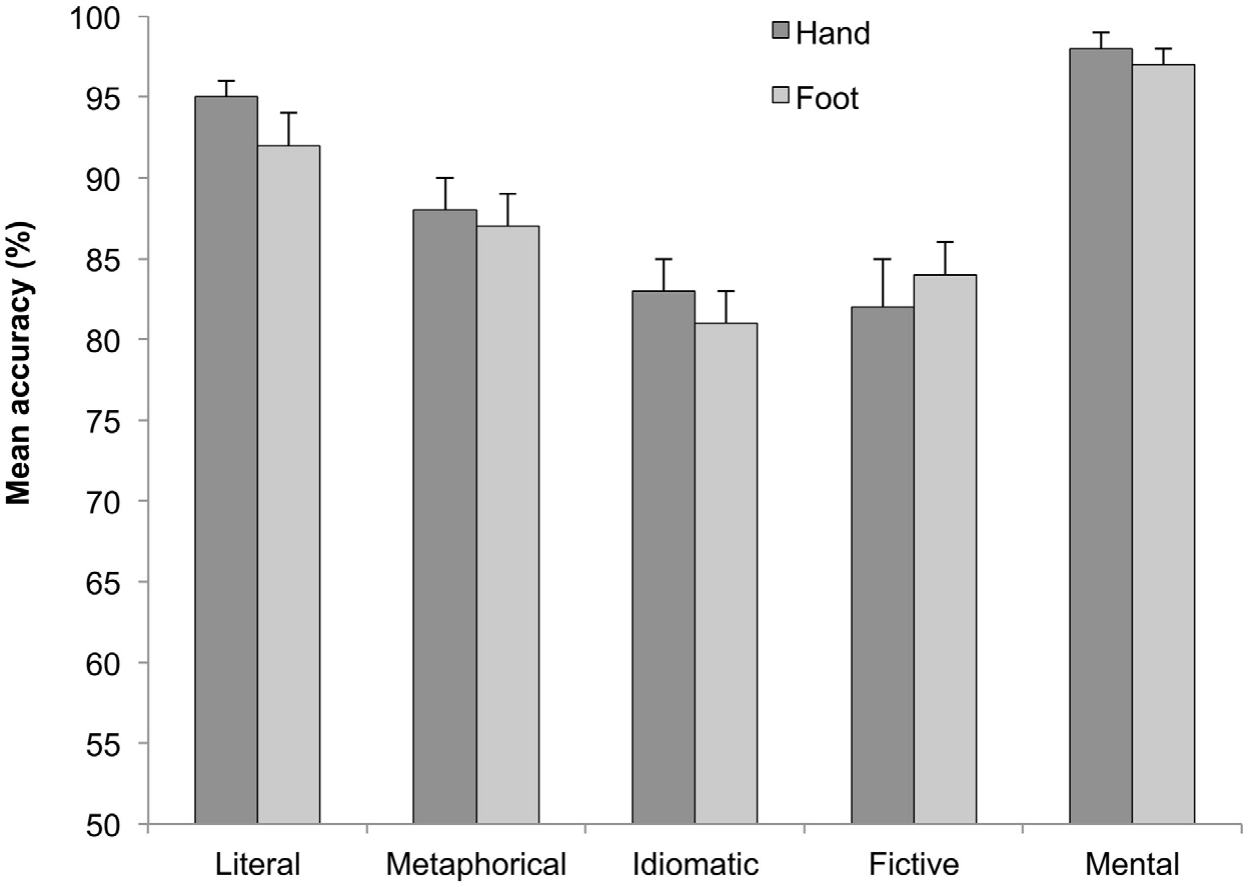
**Mean percentage of correct responses emitted with hand (dark gray bar) and foot (bright gray bar) effectors in literal, metaphorical, idiomatic, fictive motion, and mental sentences**.

One participant was discarded due to low accuracy (55%). The mean response times (RTs) to correct answers and the accuracy proportions in the different conditions are plotted in Figures 1 and 2. RTs exceeding ±2 *SD* were eliminated (2.1%). The mean error rate was 2.8%. The RTs of correct responses and the accuracy proportions were analyzed employing mixed-effects models (Baayen et al., 2008). The dependent variable was dichotomous in the accuracy analysis, hence a logistic model was applied (Jaeger, 2008). Two factors were considered: Sentence type (literal vs. metaphorical vs. idiomatic vs. fictive motion vs. mental sentences) and Effector (hand vs. foot). Participant and item were introduced as crossed random effects. Models were tested using the *lmer()* function of the *lme4* package of R, and models comparisons were assessed using the *anova()* function which calculates a Chi-square test for evaluating the difference between models goodness of fit, following Baayen's (2008) procedure. Finally, the *F* statistic and *p* value were obtained with the *anova()* and the *df()* functions, respectively.

Effects were evaluated one by one on the basis of likelihood ratio tests: those whose inclusion did not increase significantly the goodness of fit of the model were removed from the analysis. The final model on correct response times showed a main effect of Sentence type (*F* = 7.08, *p* < 0.01), and a Sentence type × Effector interaction (*F* = 3.16, *p* < 0.02). Table 2 illustrates the model parameters. As can be seen from the last-but-third line in the Table, the interaction is motivated by the fact that foot responses were quicker than hand responses, but only in literal motion sentences (1701 ms vs. 1788 ms, see also Figure 1). The final model conducted on mean accuracy proportions only showed a significant main effect of Sentence type (*F* = 20.99, *p* < 0.0001).

**Table 2:** **Fixed effects in the final model on correct response times**.

**Fixed effects**	**Estimate**	**Std. error**	***t*-value**	***p*-value**
Intercept	7.45	0.03	243.40	0.0001
Sentence type: idiomatic	0.05	0.03	1.61	0.11
Sentence type: literal	0.02	0.03	0.64	0.52
Sentence type: metaphorical	0.05	0.02	2.11	0.04
Sentence type: mental	-0.03	0.03	−1.09	0.28
Effecton: foot	0.01	0.02	0.37	0.71
Effecton: foot × sentence type: idiomatic	-0.01	0.02	−0.30	0.76
Effecton: foot × sentence type: literal	-0.06	0.02	-2.74	0.006
Effecton: foot × sentence type: metaphorical	0.01	0.02	0.41	0.68
Effecton: foot × sentence type: mental	0.004	0.02	0.19	0.85

We also considered whether some of the semantic characteristics of our motion sentences, notably concreteness, figurativeness (and idiom familiarity and semantic transparency for idiomatic sentences), affected foot response times. Neither concreteness nor figurativeness ratings significantly correlate with the foot response times of any of the motion sentence types. Idiom familiarity and semantic transparency did not significantly correlate with foot response times either (Pearson *r* values all below statistical significance with α = 0.01).

## Discussion

Overall the present results suggest that motor activation is detectable at the end of the sentence only when the sentence conveys a literal change of location. It should be recalled that in Cacciari et al. (2011), the highest motor excitability (as reflected by the largest MEPs) was recorded on literal motion sentences. In contrast to our TMS study (Cacciari et al., 2011) on the same experimental materials, we did not find any trace of motor activation in fictive and metaphorical motion sentences. In contrast to our hypothesis, but as in Cacciari et al. (2011), we did not find any motor activation for idiomatic sentences regardless of the time left for responding and of the full sentence presentation format.

We found that foot responses to literal motion sentences were faster than hand responses. One might wonder whether this may reflect the fact that in general foot responses are faster than hand responses. However, if this was indeed the case, we should have found faster foot responses in all sentence types. But this did not occur: in fact, foot response times were even slightly longer than hand responses in metaphorical motion and mental sentences (27 and 11 ms, respectively) and exactly as long as hand response times in fictive motion sentences. This questions the possibility that foot responses may be in general quicker than hand responses. It should also be noted that studies using hand vs. foot responses showed that typically hand responses are faster than foot responses (e.g., Buccino et al., 2005).

Another possible concern is why some previous studies found an effector congruency effect at the end of action-related concrete and abstract sentences (e.g., Glenberg and Kaschak, 2002) and we find this effect only in literal motion sentences. Some methodological differences may account for this inconsistency: for instance, in Glenberg and Kaschak the sentences were shorter than ours, had an abstract but literal meaning (or at least their potential figurativeness was not controlled for). In our study the literalness/figurativeness dimension was carefully controlled for so that we had either literal or non-literal sentences but not a *mixed bag* of stimuli. In fact, as noted in a recent review article by Willems and Casasanto (2011), whether motor areas are activated when participants understand non-literal uses of action-related language has produced mixed results also because *these studies have tested a mixed bag of non-literal language: action metaphors, action idioms and non-action verbs derived (diachronically) from action verbs* (p. 7). Then, the embodied literature mostly used a go/no go variant of the sentence sensibility task instead of a 2-choice variant, as in the present study. Recent studies (e.g., Gomez et al., 2007) suggested that measuring response times using go/no go vs. 2-choice variants of a task may produce different results due to different response criteria and/or decisional processes at work in the two variants.

In sum, the present results suggest that the less literal was the change of location conveyed by the sentences, the more motor activation faded away as time passed such that, at the end of the sentence, motor resonance was alive only in the *strongest* case: sentences conveying an actual action performed by an animate agent. The results of our TMS study reflected the motor excitability evoked by motion sentences while sentential processing was still unfolding or had just finished. Although we know from several studies (for overviews, see Glenberg and Kaschak, 2002; Fischer and Zwaan, 2008) that effector congruency effects reflect the involvement of the motor system, at a purely behavioral level this effect may register a less direct brain response to action-related sentences than when motor excitability is directly recorded with TMS (and at short lags, in our study) or MEG (for a discussion, see Boulenger et al., 2012). In other words we cannot exclude that motor activation indeed occurred at the verb in all motion sentences (Zwaan and Taylor, 2006) and then decayed as a function of the strength of the action-related meaning of the sentence until being in most cases undetectable at the end of the sentences.

The possibility that motor system become active at the verb position for then decaying as a function of the strength of the semantic motion component of the sentence is compatible with the *Linguistic Focus Hypothesis* (Zwaan and Taylor, 2006). According to this hypothesis, motor activation may be short-lived at a sentential level in that it may not extend beyond action-specifying verb. Hence, it may progressively fade away after the verb for being undetectable when subjects emit the sensibility judgment at the end of the sentence. The idea that motor activation may be short-lived is also consistent with previous studies, for instance with the MEG study by Pulvermüller et al. (2005) where it was shown a short-lived language induced motor activity at around 150 ms. As Nazir et al. (2008) pointed out, it can be the case that action words used in non-literal ways, as for instance in *The cash machine swallowed his credit card*, may engage *cortical motor regions during lexical access for the word “swallow” but probably not during subsequent access to the meaning implied by the sentence* (p. 940).

Non-literal motion sentences did not convey any actual action. They represent a typical case of abstract meanings conveyed by verbs that, in other linguistic contexts, may instead denote a concrete action. As Kousta et al. (2010) noted, it is not obvious how an embodied account can be valid for abstract meanings. One possibility is to presuppose that all non-literal motion sentences originate from embodied conceptual metaphors (Gibbs, 2006). However, it is still controversial whether conceptual metaphors are indeed part of our online understanding of non-literal language (for an extensive discussion, see Katz et al., 1998), how they are acquired and mentally represented and whether they are fundamental in the development (and representation) of abstract concepts and word meanings (Kousta et al., 2010). Then, even assuming that upon reading an idiomatic motion sentence one activates the embodied simulation corresponding to the underlying conceptual metaphor (Gibbs, 2006), the processing mechanism underlying such a univocal mapping are not yet spelled out. For instance, let us take Italian idioms such as, for instance, *scendere dal pero* (*climb down the pear tree*, i.e., abruptly discover the truth), *andare a monte* (*go to mount*, i.e., fail) or *venire alle mani* (*come to the hands*, i.e., fight). These are semantically opaque idioms taken from the experimental stimuli of the present study. How can we identify the corresponding underlying conceptual metaphors and map them onto the specific sentential context? In any case, if the semantic structure of the underlying conceptual metaphors (if any) had played any role in determining foot response times, we should have found a significant correlation between semantic transparency and response times, but this was not the case.

What are the implications of the present results? First, they showed that the engagement of the motor system in the semantic processing of sentences with motion verbs is constrained by the linguistic context in which the verb occurred. Of course, this holds true if we assume that the behavioral task we employed implies motor system activation, as previous studies showed (e.g., Glenberg and Kaschak, 2002). Our results confirm that motor cortex did not respond to motor verbs indiscriminately replicating part of the results previously observed in TMS studies on the same experimental materials (Cacciari et al., 2010, 2011; see also Willems and Casasanto, 2011 for further evidence). This undermines the generality of the claim of a causal contribution of motor activation to the semantic processing of motion sentences. Our results also suggest the possibility that the more time passed from the presentation of the motion verb, the more motor activation faded away. Finally, our results favor the idea that for comprehending abstract concepts (as those conveyed by non-literal sentences) linguistic information is crucial and certainly more relevant than sensory-based information. In fact, idiomatic motion sentences were well-understood by participants despite the fact that no motor activation occurred, as shown by both the TMS and the present study. In sum, definitively the activation of motor or sensory information may contribute to but definitively not replace the semantic analysis of a sentence.

### Conflict of interest statement

The authors declare that the research was conducted in the absence of any commercial or financial relationships that could be construed as a potential conflict of interest.

## Appendix

Italian examples of sensible sentences with word-by-word English translations:

*Literal motion:* Claudia salta la corda in cortile (Carla jumps the rope in the yard).
*Metaphorical motion:* Lo studente salta da un libro all'altro (The student jumps from a book to another one).
*Fictive motion:* La ferrovia salta quel paese isolato (The trains jumps the isolated village).
*Idiomatic motion:* Alice salta di palo in frasca sempre (Alice jumps from pole to branch always).
*Mental Verb:* Il padrone garantisce un aumento di stipendio (The owner guarantees an increase in the salary).
*Literal motion:* Guido esce dall'aula magna universitaria (Guido goes out from the assembly hall).
*Metaphorical motion:* La signora esce dai pensieri del marito (The lady goes out from the husband thoughts).
*Fictive motion:* La pista esce dal confine italiano (The trails goes out from the Italian border).
*Idiomatic motion:* Il politico esce di scena velocemente (The politician goes out from the scene quickly).
*Mental Verb:* Riccardo capisce la soluzione del quiz (Riccardo understands the solution of the problem).
